# Drug Addiction: From Neuroscience to Ethics

**DOI:** 10.3389/fpsyt.2018.00595

**Published:** 2018-11-19

**Authors:** Michele Farisco, Kathinka Evers, Jean-Pierre Changeux

**Affiliations:** ^1^Centre for Research Ethics and Bioethics, Uppsala University, Uppsala, Sweden; ^2^Science and Society Unit, Biogem, Biology and Molecular Genetics Institute, Ariano Irpino, Italy; ^3^Collège de France and Institut Pasteur CNRS, Paris, France

**Keywords:** addiction, ethics of addiction, unaware processing, opioids epidemics, drugs addiction

## Abstract

In the present paper, we suggest a potential new ethical analysis of addiction focusing on the relationship between aware and unaware processing in the brain. We take the case of the opioids epidemics to argue that a consideration of both aware and unaware processing provides a more comprehensive ethical framework to discuss the ethical issues raised by addiction. Finally, our hypothesis is that in addition to identified Central Nervous System's neuronal/neurochemical factors contributing to addictive dynamics, the socio-economic status plays a causal role through epigenetic processes, originating the need for additional reward in the brain. This provides a strong base for a socio-political form of responsibility for preventing and managing addiction crisis.

## Introduction

Even if, as recognized, among others, by the World Health Organization and by the American Psychiatric Association, addiction can be described as a chronically relapsing brain disorder which shares the same brain pathways of reward systems, there is a growing discussion about whether addiction should be understood as a brain disease/disorder or as resulting from a non-pathological brain dynamics/development (1–8). The point being whether addiction results from a pathological neurobiological disorder or rather from a brain dynamics eventually manifesting in addicted behavior. In both scenarios addiction is correlated with some changes in brain systems, particularly in networks mediating experience and anticipation of reward, perception and memory, and cognitive control (7), but the point at stake is whether such changes should be regarded as pathological or rather as brain developments caused by particular biological, psychological and environmental factors. These two alternative views result in different interventions: if addiction is a neurobiological pathology medication is the only way to treat it; if addiction is a non-pathological brain development, then changing the factors causing it would restore a non-addicted brain state. We acknowledge the controversy around the definition of addiction as a disease, as well as, its potential impacts on different levels (from diagnosis to prognosis, from ethics to policy definition). Yet, as we will see in details in the following, we think that new scientific perspectives on brain development and on consciousness/non-conscious processing relationship offer the possibility of conceptualizing addiction beyond a dualistic interpretation of disease and dynamical models.

Beyond the abovementioned scientific controversy, addiction has emerged as one of the most compelling emergencies of contemporary society. Many factors contribute to making addiction a complex and multifaceted issue, the analysis of which requires the contribution of different fields.

Several scientific (e.g., from neuroscience to medicine) and social analyses of addiction have been produced in recent years (4, 9). However, the ethical discussion seems to be more limited and it is mainly focused on normative and practical issues (10, 11), i.e., on the regulatory and practical questions related to the off-label abuse of opioid medication. An ethical analysis of the factors leading to addictive behaviors and, specifically, of the responsibility for such behaviors seems lacking. This paper aims to contribute to this kind of ethical analysis on the basis of neuroscientific data about information processing in the brain. In particular, we focus on the role of external influences on unaware processing (also referred to as unconscious, preconscious, or non-conscious processing) and its driving role in addictive behavior.

We will argue that in addition to the central nervous system's neuronal/neurochemical bases of addiction, particularly to opiate crisis, socio-economic status modulates through non-conscious processing what can be described as the person's subjective “global well-being,” raising the need for additional rewards in the brain. In the light of the impact of external factors, we argue that some people are particularly vulnerable to a sort of alienation by the political-socio-economical capitalistic system, and that this stressful condition, which has both aware and unaware components, is one of the main causes of addiction.

In fact, different factors contribute to define the complexity of addictive behavior, which includes both physiological/pharmacological and psychological/social components. As a result, the subject acts as conditioned by both aware and unaware drives. Aware and unaware levels, their respective interaction, and the impact of external factors should be taken into account when attempting to provide a more adequate ethical analysis of addiction.

The case of opioid epidemics is highly illustrative in this respect. It clearly shows how addiction is affected by both internal and external factors, e.g., physiological, psychological, and environmental mostly social.

## A contemporary epidemics

### Opioids abuse data

Data shows that opioids abuse has increased significantly in the last decades just in the US (9, 12): the rate of opioid addiction affected about 2.5 million adults in 2014, while in 2016 91.8 million of US civilian used prescription opioids, and 11.5 million misused them (13).

Media coverage of the opioids addiction has been growing in recent years. In the last few years, *Time* magazine devoted two covers to the abuse of opioids painkillers [June 2015 (14) and March 2018 (15)]. Yet many other communication media reserve significant attention to the phenomenon almost every day: just to give some recent examples, *CNN* website recently published the data of the fast increase of opioid crisis (16); *The Washington Post* published a tough story of life devastated by opioids (17); and *The New York Times* described the stories of the children of addicted mothers (18).

Increases in opioids abuse are related to increases in therapeutic opioids prescription (12). The main claimed reason for those prescriptions is chronic pain, whose prevalence among adult Americans is between 30 and 40% (19). Opioid medication is now the most prescribed medication in the US (20).

As recently outlined by Volkow and McLellan, two facts seem indubitable: first, opioid analgesics are widely distributed and improperly used, resulting in a high number of overdose deaths and addictions; second, the main source of opioids distribution is physicians' prescriptions (9, 21, 22).

These two facts raise important ethical issues that should be addressed. The main issue is the responsibility, i.e., the original cause, for such a widespread improper use of opioids that many people define as an epidemic. What are the real causes leading to an improper use of opioids?

### The legal use of opioids as pain killers

Different causes can be related to the actual massive use of opioid analgesics. One of the main factors is the perception of pain as a negative experience to be cured and eventually eliminated. More specifically, the Joint Commission on the Accreditation of Healthcare Organizations (JCAHO), incorrectly assuming that clinical use of opioids rarely generates addiction, reported that effective narcotic analgesics were wrongly *not* used in US because of an irrational fear of addiction (23). A strong focus on pain management by opioids ensued, accompanied by pharmaceutical companies marketing.

The pro-opioids for pain management movement gained strength from this report and had a large impact on the public, contributing to subtly change the perception of the medical use of opioids. As a consequence, the prescription of opioid analgesics increased considerably (24). The problem is that the number of non-prescribed, diverted use of opioids seems to be proportionally related to the number of prescriptions (12); the transition from medication to addiction being something subtle and not consciously perceived by the affected subject (25). Addiction starts as an unaware process: only in subsequent stages does the subject become aware of her/his addiction and takes the drug knowing it is a drug.

An important reason for the continued use of opioids is that they are prescribed by physicians, hence perceived as either less or not dangerous at all (26). This fact points not just to the physicians' responsibility (both as causal role and accountability) but also shows the influence of implicit biases on the resulting addiction behaviors. In general, the doctor is implicitly seen as an ethically normative actor, someone who clearly makes the difference between licit and illicit behaviors (27). Moreover, because opioids are legally prescribed as painkillers there is a tendency to regard them as safer drugs (28). The impact of these implicit biases on the final addiction behavior raises the issue of socio-political responsibility: legally allowing the prescription of opioids medication is not neutral with regard to the perception of its risks. In fact, we think that, in the light of both aware and unaware influences that can be exercised on the final users, legally allowing the prescription and subsequent use of opioids risks to be a way to endorse them.

Among the personal reasons to continue opioids use, the most important seems to be their role as a response to life stressors or as a means of self-medicating psychological issues, effects of trauma, or emotional pain. Other, minor reasons include normalization (e.g., to not feel uncomfortable), increased energy, boredom, enhanced sexual intimacy, self-blame/addictive personality (12). Co-morbidity of opioids addiction with underlying psychiatric disorders is quite high in prescribed opioid addicted subjects. The risk of addiction has also genetic and developmental roots: in particular adolescents are more prone to develop addiction (29, 30).

The above suggests that addiction has a strong basis in the brain on different levels of consciousness, aware, as well as, unaware. For this reason we think that an ethical analysis of addiction purporting to highlight the reasons behind it must include a specific focus also on the lower levels of consciousness, not connected to awareness.

## The pharmacological impact of addiction on awareness

While different theoretical models of conscious processing have been elaborated on the basis of cognitive neuroscience [e.g., the Global Neuronal Workspace (31, 32)], little attention has been dedicated to the pharmacology of conscious experience (33). There is evidence that self-awareness, an important component of conscious experience, is determined by a paralimbic circuitry of 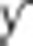 synchrony regulated by GABAergic interneurons under the control of acetylcholine and dopamine. Accordingly, specific chemical agents and their respective balance modulate awareness.

Specifically, self-awareness has been shown to be linked to hemodynamic activity in a medial paralimbic circuitry involving medial prefrontal/anterior cingulate, medial parietal/posterior cingulate, and subcortical regions, associated with the lateral parietal cortex, typically the angular gyri, and insula (33). On the basis of experiments with transcranial magnetic stimulation (TMS), Changeux and Lou conclude that the paralimbic circuitry plays a causal role with regard to self-awareness. Interestingly 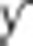 synchronization in the paralimbic circuitry regions increases proportionally with self-processing. The conclusion by Changeux and Lou is that the paralimbic synchronization enables unity of consciousness through coherence of serial conscious experiences (e.g., self-control) by acting as their common neural path (33). In other words, 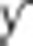 synchronization of paralimbic regions plays a crucial role in self-awareness and self-control. 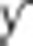 synchronization is regulated by GABAergic interneurons, which are affected in particular by two neurotransmitters: acetylcholine and dopamine.

Behaviorally, addiction may be described as the result of the loss of or the serious impairment of self-control, decision-making, and emotion processing by the subject, where an initially voluntary substance use or behavior gradually becomes compulsive (33, 34). More specifically, three stages of addiction have been identified (2): preoccupation/anticipation; binge/intoxication; withdrawal/negative effects. Importantly, these three stages feed into each other reinforcing an addiction cycle.

At the neurophysiological and neurobiological levels, addiction causes the impairment of the paralimbic circuitry that we have seen to be critical for self-awareness and self-control. Consequently, addiction results in a pharmacological disorder or chemical impairment of conscious self-control and self-regulation through the impairment of paralimbic medial circuitry normal function (33, 35). The abovementioned three stages are correlated with changes occurring in three brain systems related to particular functions: preoccupation/anticipation is correlated with changes in the prefrontal cortex, which underlies executive function; binge/intoxication is correlated with changes in the basal ganglia, which underlies incentive salience; and withdrawal/negative effect is correlated with extended amygdala, which underlies negative effect withdrawal (2).

In short, in cases of addiction there are important changes in the brain reward and stress mechanisms, underlying the passage from impulsive to compulsive behavior and from positive to negative reinforcement. Importantly, addiction affects, in particular, the dopaminergic system, which we have seen plays an important role in modulating self-awareness and self-control. In the end, addiction causes a pharmacological disconnection from top-down GNW processing, i.e., a moving from self-awareness to unchecked goal-directed actions (36).

In other words, addiction causes the disruption of the chemical balance critical for self-awareness and self-control, causing a vicious circle for which the dependence from the substance constantly increases (33).

## Ethics of addiction

In addition to the neurochemical bases of consciousness and of the addiction's impact on it described above, socio-economic and ecological contexts play an important role in addiction insofar as they have a significant impact on the brain aware and unaware processes. The connection between the brain and its living contexts gives us new tools for detecting ethical responsibility for addiction.

An important scientific theory for exploring the connection between brain and external world is the epigenetic theory of neuronal development, which promises to help us illustrating addiction dynamics as well.

### Synaptic epigenesis and the internalization of the socio-cultural environment during development

As recently summarized elsewhere (37), recent advances in neuronal epigenesis studies reveal a deep relationship between the brain and its environment, including social and cultural contexts (38). There is evidence that because of this interaction, an active epigenetic selection of neuronal networks results in the internalization of the cultural and ethical rules prevalent in the social community to which the child and her/his family belongs (39). Arguably, this internalization is mostly implemented at the unaware level and importantly contributes to shaping the brain's architecture on all levels of conscious processing.

The epigenetic theory of neuronal development together with other studies about the intrinsic predisposition of the brain to interact with the world (40) suggest a reciprocal causality between the brain and its external environments, and a mutual epistemic relevance in understanding the two realms (biological vs. socio-cultural) (37). Specifically, understanding the brain requires reference to the experiences and social structures that shape it, and knowledge of the brain is also relevant to understanding the development of those social structures (41, 42).

From the above we can infer that the brain is not a closed, self-referential information device or a simple input-output machine, but rather a plastic and interacting organ shaped by a panoply of factors, including biological, experiential, and social causes.

#### The quality of life and general welfare

One of the reasons for addictive opioids consumption is likely the lack of well-being understood in its widest sense, that is including both psychological and physical components. As is characteristic of any addictive behavior, an initial need for treating an undesirable condition (e.g., physical pain) is subsequently replaced by the urge to “feel high” and then the need to oppose the withdrawal symptoms. Significantly, the transition from using opioids as painkillers to using them addictively takes place without the subject being aware of it: it seems like the user loses control of what s/he intakes and of the true reasons why she/he intakes it (25).

The critical component in this addiction dynamics is how “being well” is conceived and consequently exposed to external influences. As seen above, recent evidence from neuroscience depicts the brain as a cognitive and emotional, spontaneously active organ, which is shaped and modulated by the interaction with the environment (43). Its cognitive and emotional actions are not limited to the aware level, nor do they result from internal factors only: environmental influences on the development of the cognitive and emotional brain, at both aware and unaware levels, are massive and even critical.

This suggests the view of “being well” as a multilevel and multidimensional condition: well-being can generally be perceived at both unaware and aware levels, and it results from different factors, both internal and external to the subject (e.g., bodily components and environmental influences). Among the factors impacting on brain development are the influences on subjective well-being coming from the socio-cultural environment, including political, cultural, and educational contexts: the information coming from these sources are internalized by the subject and contribute to shaping his personal aware and unaware well-being. The relevance of external factors in shaping individual actions raises the issue of social responsibility, if not in ethical terms, at least in terms of public policy.

It is significant that the opioids addiction described above primarily if not exclusively affects advanced industrial countries, and in particular the US. Opioids consumption is not new in society: for instance, they were abundantly used in ancient societies. Anyway, even if always questionable, the use of drugs usually had a different socio-political meaning and function, and they were almost systematically used in social and religious rituals under a very stringent control, for instance by shamans. Today, the reason, why to use drugs is different: coping with life stressors and looking for well-being seems to be among the main reasons leading to addictive opioids consumption. Such addiction is prevalently affecting advanced industrial countries, so that it is reasonable to infer that these societies may be affected by a general feeling of dissatisfaction, which emerges as a psychological, social, political, and ethical issue. Even further, such dissatisfaction might be at the root of the search for enhancement, including brain enhancement, which has been one of the priorities of US neuroscience research, e.g., in military research (see https://www.darpa.mil/program/targeted-neuroplasticity-training).

This is comparable with the general tendency lately spread in Europe and also the US to think about the need to go over present humans, like in the transhumanist philosophy, which aims at purifying humankind from its poor present state (44), finally emerging as a sort of secular eschatology. A main issue with important ethical implications raised by this view is the definition of the quality of life standards from which we can infer whether our status is good or bad. The internalization of such standards, which result from several external sources and subsequently affect how the subject consciously thinks and acts, is arguably happening mainly below the level of awareness.

Finally, we think that the abovementioned feeling of dissatisfaction has its roots in the value system of Western societies, dominated by a capitalist worldview according to which personal success is measured by economic and financial success. The Western value system grounded on competition vs. cooperation is arguably one of the causes of the life condition leading to addictive consumption of drugs. It is a remarkable phenomenon that the recent dramatic increase of opiates overdose casualties closely follows that of income inequality in the US (45). Again, from an ethical point of view, an alternative to this value system is possible, for instance in stressing the priority of cooperation over competition, and community and esthetic pleasure as a social value (46).

#### The aware feeling of pain

The original reason why opioids medication was massively introduced in the healthcare system was the need to manage and treat pain, seen as something to be eliminated. This raises the question of the definition of pain. This is a scientific issue with important ethical implications. In fact, we can generally describe pain as an evolutionary warning system, a sort of safety device making subjects consciously aware of a danger without necessarily being aware of the causes of the pain. Accordingly, pain acts as a homeostatic behavioral regulator: it is both an emotion (i.e., interoceptive knowledge of physiological condition) and a behavioral motivation originating from the need to maintain homeostasis (47–49). Homeostasis can be described as a dynamic and ongoing process maintaining an optimal balance in the physiological condition of the body for its survival (50).

If so, from an evolutionary point of view pain is not a negative but rather a necessary phenomenon. It is reasonable to think that without pain the chances of survival of humankind, and of any animal species, would be much lower. The inherited condition known as congenital insensitivity to pain confirms the necessity of pain for surviving: people affected by such disorder frequently die prematurely due to complications of trauma and injuries (51).

The issue to address, then, is whether and to what extent it is worth to manage pain simply by silencing or abolishing it. Completely suppressing pain would mean eliminating a system of physiological feedback regulation between the subject and the outside world. In other words, the capacity for experiencing pain is necessary for the survival of living organisms. The individual fitness would be consequently affected if pain was simply removed. The reason is that both positive and negative rewards are necessary for an appropriate evaluation of the external world.

This is not to say that all pain is “valuable,” or that not being able tolerating pain is unjustifiable or in any way worthy of stigmatization. There may be conditions of pain that the subject cannot—and should not be requested to—endure [which they are is open to controversy, e.g., particularly debilitating chronic pain, or end-of-life conditions. See for instance the Final Report by the President's Commission on Combating Drug Addiction and the Opioid Drugs (52)]. In these cases, the use of painkillers, including exceptionally opioids, would indeed be both medically and ethically justified. Our argument directs itself exclusively against the use of opioids, where this is not—or not demonstrably—the case, recognizing that the drawing of this limit can be a difficult challenge. Moreover, we stress also the ethical need to enhance the search for alternative, non-addictive painkillers, including non-addictive opioids like recently suggested by Severino et al. (53).

#### The responsibility of drug companies and medical doctors

The opioids epidemics was partly initiated by the pharmaceutical companies which developed a very potent opiate analgesic without warning against—or at worst even denying—the risk of addiction. A pro-opioids campaign was initiated on the basis of the erroneous assumption that the use of this medication was free of any risk of addiction (23). These companies put great social pressure on medical doctors and this massively affected both public opinion and professional standards. The influence that pharmaceutical companies have on society and how they impact public opinion and professional choices is ethically problematic and requires a specific analysis.

First, the reasons behind such influence should be scrutinized: what is the aim of drug companies' battle in favor of opioids medication? Is it patients' interest or rather their own (economic) interest, or a combination of both?

The behavior of medical doctors should also be critically assessed. If even when they know about the risk of addiction physicians still choose to prescribe opioids, such choice may be ethically problematic. It is true that it is not easy for the doctor to choose how to best help the patient and maybe the sole cost-benefit analysis is insufficient *per se* to solve the dilemma. The risk of addiction may be outweighed by the benefit of pain-relief, but this is a difficult medical and psychological evaluation that must be done with great care, not least since there may be quite large differences between different individuals concerning the best possible treatment. Moreover, if the mere fact that a medical doctor prescribes a medication makes lay people believe that such medication is ethically unproblematic, this means that what the doctor says is not neutral but has important consequences both in terms of opinions and in terms of action, mainly influencing them at the unaware level. This fact is ethically significant.

An ethical warning informed by the scientific data about aware and unaware brain processes should be part of both drug companies' policies and medical doctors' professional skills, and relevant tools should be implemented to increase understanding of these topics.

#### A look forward

The discussion above suggests that a number of considerations should be taken into account in the search for a feasible and effective strategy to manage addiction.

In the first place, any attempt to cope with addiction should start from the relevant scientific knowledge, particularly from the neuroscience of the involved aware and unaware processes. In fact, the dynamics of addiction includes both aware and unaware components: as illustrated by the case of opioids, at the beginning the subject consciously chooses to take the drug to alleviate a negative experience (e.g., pain). This (apparently) fully aware decision is partly affected by unaware dynamics that are beyond direct subjective control. Whether undergoing pain is taken to be a negative experience and what amount of pain can be tolerated depend in part on external information (e.g., from professional organizations, educational actors, social media) that eventually becomes interiorized and affects subjective behavior at the unaware level. Thus, when the subject asks the doctor for an opioid prescription, and she/he consciously starts to take the medication, her/his behavior is already subtly conditioned and eventually guided by both aware and unaware drives until an addictive use of the substance is established. As emerges from first person accounts (12), initially addicted subject has no knowledge of being addicted, she/he is not aware. The realization of addiction comes only at a later stage, when she/he continues to take the drug knowing that it is an addiction and unable to stop using it because of withdrawal and other negative symptoms.

In the end, it is necessary to be aware of this continued oscillation between aware and unaware drives, which denote different psychological, neurological, and pharmacological processes in the brain. Since neuroscience is providing increasing knowledge of these processes, management strategies should consider both the aware and unaware brain. Of course, such strategies can be implemented in different ways, e.g., through a direct pharmacological approach or through an indirect approach aiming at influencing the brain by altering external environmental conditions, including cultural and social institutions. In particular, considering that brain development is particularly sensible to external inputs for about 20 years after birth (43), the experiences during this period of time, especially familiar and educational conditions, play a crucial role in exposing the subject to the risk of addiction.

## Conclusion

An ethical framework for a balanced analysis of addiction should take into account emerging neuroscientific data about aware and unaware processes involved. In order to clarify the ethical responsibility of the final user, of the medical doctors, and of the pharmaceutical companies, and to suggest a strategy for an ethically sound management of addiction it is necessary to include different levels of conscious processing in the brain, not only awareness, and to outline the critical role they play in addiction behavior, the extent to which external influences shape it, and the possibility to take care of it. Thus, we suggest that an ethics of addiction (i.e., an ethically sound treatment of addiction) importantly requires taking due care of the brain also below the levels of awareness.

In short, our argument rests on the following:

1. Medical: Addiction causes the disruption of the chemical balance critical for self-awareness and self-control, resulting in a pharmacological impairment of awareness and causing a vicious circle for which the dependence from the substance constantly increases.
2. Scientific: Non-conscious brain processes are massively influenced by socio-economic and ecological factors.
3. Psychological: Addiction is mainly dependent from non-conscious brain processes, i.e., from loss of conscious control.
4. Ethical: Given the scientific and psychological factors mentioned, the socio-economic and ecological contexts are highly relevant to addictive dynamics, especially through the influence they have on unaware brain processes.

Finally, our hypothesis is that in addition to identified Central Nervous System's neuronal/neurochemical factors contributing to addictive dynamics, the socio-economic status plays a causal role through epigenetic processes, originating the need for additional reward in the brain. For this reason, we consider addiction to be, *in addition* to a medical and mental disorder, *also* a social disorder.

This provides a strong base for a socio-political form of responsibility for preventing and managing addiction crisis.

## Author contributions

MF wrote the manuscript and was responsible for general ideas. J-PC contributed to revising and developing ideas. J-PC and KE commented on previous versions of the manuscript and helped in developing lines of argument.

### Conflict of interest statement

The authors declare that the research was conducted in the absence of any commercial or financial relationships that could be construed as a potential conflict of interest.
